# Dopaminergic Neuronal Loss and Dopamine-Dependent Locomotor Defects in Fbxo7-Deficient Zebrafish

**DOI:** 10.1371/journal.pone.0048911

**Published:** 2012-11-02

**Authors:** Tianna Zhao, Herma Zondervan-van der Linde, Lies-Anne Severijnen, Ben A. Oostra, Rob Willemsen, Vincenzo Bonifati

**Affiliations:** Department of Clinical Genetics, Erasmus MC, Rotterdam, The Netherlands; Emory University, United States of America

## Abstract

Recessive mutations in the *F-box only protein 7* gene (*FBXO7*) cause PARK15, a Mendelian form of early-onset, levodopa-responsive parkinsonism with severe loss of nigrostriatal dopaminergic neurons. However, the function of the protein encoded by *FBXO7*, and the pathogenesis of PARK15 remain unknown. No animal models of this disease exist. Here, we report the generation of a vertebrate model of PARK15 in zebrafish. We first show that the zebrafish Fbxo7 homolog protein (zFbxo7) is expressed abundantly in the normal zebrafish brain. Next, we used two zFbxo7-specific morpholinos (targeting protein translation and mRNA splicing, respectively), to knock down the zFbxo7 expression. The injection of either of these zFbxo7-specific morpholinos in the fish embryos induced a marked decrease in the zFbxo7 protein expression, and a range of developmental defects. Furthermore, whole-mount *in situ* mRNA hybridization showed abnormal patterning and significant decrease in the number of diencephalic *tyrosine hydroxylase*-expressing neurons, corresponding to the human nigrostriatal or ventral tegmental dopaminergic neurons. Of note, the number of the *dopamine transporter*-expressing neurons was much more severely depleted, suggesting dopaminergic dysfunctions earlier and larger than those due to neuronal loss. Last, the zFbxo7 morphants displayed severe locomotor disturbances (bradykinesia), which were dramatically improved by the dopaminergic agonist apomorphine. The severity of these morphological and behavioral abnormalities correlated with the severity of zFbxo7 protein deficiency. Moreover, the effects of the co-injection of zFbxo7- and p53-specific morpholinos were similar to those obtained with zFbxo7-specific morpholinos alone, supporting further the contention that the observed phenotypes were specifically due to the knock down of zFbxo7. In conclusion, this novel vertebrate model reproduces pathologic and behavioral hallmarks of human parkinsonism (dopaminergic neuronal loss and dopamine-dependent bradykinesia), representing therefore a valid tool for investigating the mechanisms of selective dopaminergic neuronal death, and screening for modifier genes and therapeutic compounds.

## Introduction

Parkinson's disease (PD), the second most common neurodegenerative disorder after Alzheimer's disease, is characterized by the progressive loss of nigrostriatal dopaminergic neurons, and the formation of alpha-synuclein-containing protein aggregates, termed Lewy bodies, in surviving neurons [1]. The molecular mechanisms underlying PD remain poorly understood, but the recent identification of rare inherited forms of parkinsonism has opened novel research avenues into the disease pathogenesis [2], [3]. Mutations in the *alpha-synuclein* (PARK1), *leucine-rich repeat kinase 2* (PARK8), and *vacuolar protein sorting 35* (VPS35) genes cause autosomal dominant forms, while mutations in the *parkin* (PARK2), *PINK1* (PARK6), *DJ-1* (PARK7), *ATP13A2* (PARK9), and *FBXO7* (PARK15) genes cause autosomal recessive forms of parkinsonism [3], [4]. Whether the mutations in the different forms of monogenic parkinsonisms converge on the same or different cellular pathways remains currently unclear. However, understanding the mechanisms of these Mendelian parkinsonisms might provide important clues into the pathways leading to the degeneration of the dopaminergic neurons, which might also be involved in the common forms of PD. For example, there are evidences of functional links between the alpha-synuclein and the ATP13A2 pathways [5], [6]. Our group characterized mutations in the *F-box only protein 7* (*FBXO7*) gene, encoding the F-box protein 7 (FBXO7), as the cause of PARK15 [7]. PARK15 patients display dramatic loss of nigrostriatal dopaminergic neurons, and they suffer from juvenile parkinsonism, with varying degrees of pyramidal disturbances. Of note, the parkinsonism displays a good response to levodopa therapy, indicating the relative integrity of the striatal neuronal circuitry acting downstream to the nigrostriatal dopaminergic defect [7], [8].

FBXO7 is a member of the F-box-containing protein (FBP) family, characterized by a ∼40-amino acids domain (the F-box). FBPs might become part of SCF (Skp1, Cullin1, F-box protein) ubiquitin ligase complexes, and play roles in ubiquitin-mediated proteasomal degradation [9]. We recently reported that two protein isoforms are expressed from the *FBXO7* gene, and that PARK15 patients display a severe depletion of the longer isoform, which normally localizes in the cell nucleus. The activity of FBXO7 in the nucleus appears therefore crucial for the maintenance of brain neurons in humans and the pathogenesis of PARK15 [10]. However, the precise function of the FBXO7 proteins remains largely unknown, and animal models of PARK15 have not been reported so far. Understanding why the loss of the function of this protein leads to PARK15 might illuminate the mechanisms of selective dopaminergic neuronal death, which could also be important for PD in general.

There is a growing interest in the use of zebrafish (*Danio rerio*) for modeling neurodegenerative diseases in vertebrates [11], [12]. High degrees of evolutionary conservation are present between zebrafish and human homologue proteins and pathways involved in neurodegenerative diseases. Furthermore, zebrafish offers advantages compared with other vertebrate models, including a rapid and external embryonic development, and availability of rapid and efficient tools for genetic manipulation [11], [12].

Here, we report the generation of a first vertebrate animal model of PARK15 by morpholino (MO)-mediated knock-down of the *FBXO7* homologue gene in the zebrafish (*zFbxo7*). The morphants display dopaminergic neuronal loss and dopamine-dependent locomotor defects, thereby reproducing pathologic and behavioral hallmarks of the human disease. This is a novel important tool for investigating the mechanisms of selective dopaminergic neuronal death, and for the implementation of high-throughput screening of modifier genes or compounds.

## Results

### Characterization of the *zFbxo7* gene in zebrafish

**Figure 1:**
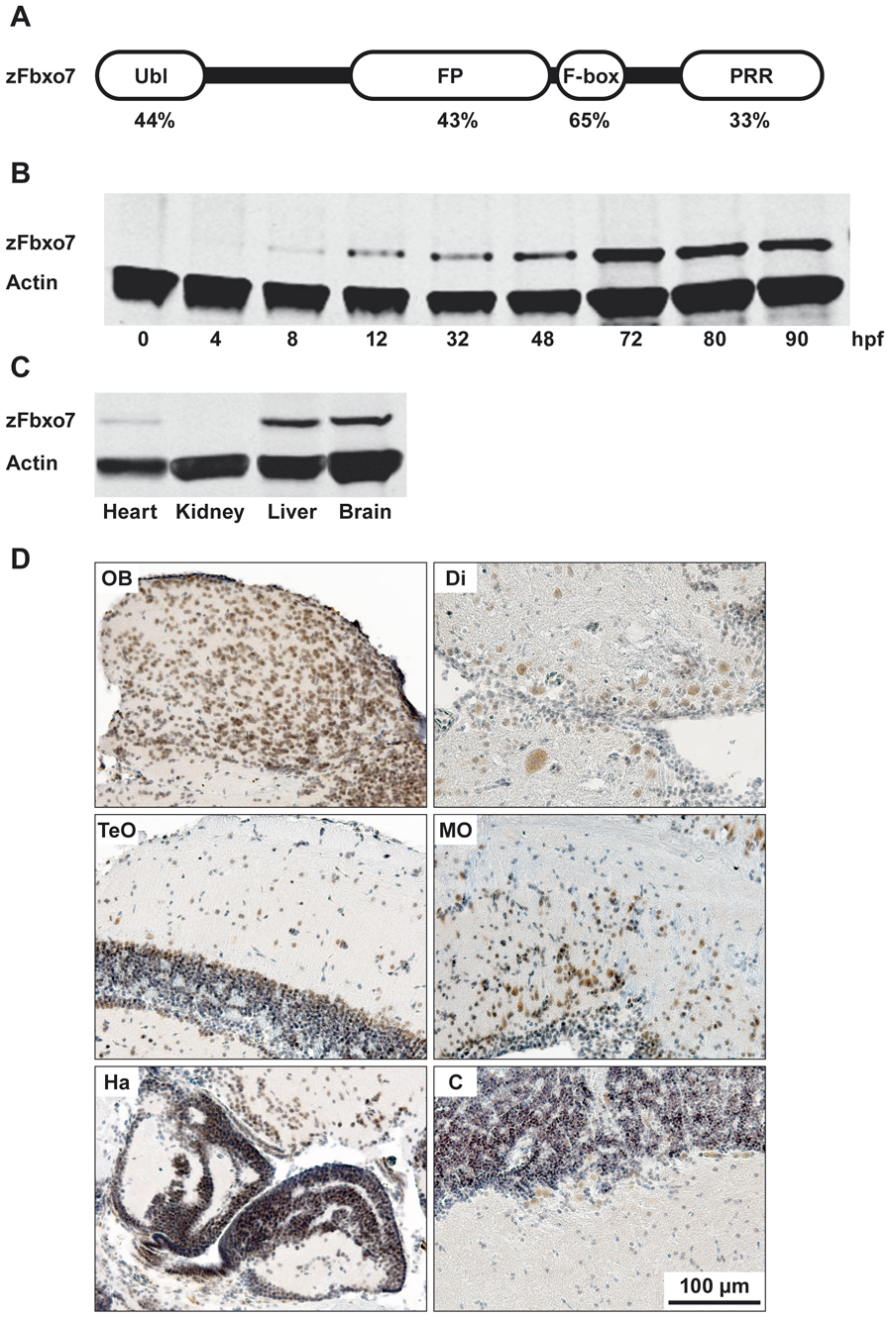
Characterization of the zFbxo7 protein in zebrafish. (A) Schematic representation of the zFbxo7 functional domains. The values underneath each domain indicate the amino acids identity between zFbxo7 and hFBXO7 (isoform1). Ubl: ubiquitin-like domain; FP: FBXO7/ PI31 domain; F-box: F-box motif; PRR: proline rich domain. (B) Western blot analysis of the zFbxo7 protein expression at different developmental stages. (C) Western blot analysis of zFbxo7 protein expression in different tissues of eight-month-old adult zebrafish. Actin was used as reference protein.(D) Immunostaining of the zFbxo7 protein in eight-month-old zebrafish brain areas. The zFbxo7 immunoreactivity is shown in brown, while the cell nuclei are counterstained in blue using hematoxylin. The following areas are shown: olfactory bulb (OB), diencephalon (Di), optic tectum (TeO), medulla oblongata (MO), habenula (Ha), and cerebellum (C). Scale bars: 100 µm.

The human *FBXO7* gene (*hFBXO7*), expresses two transcripts (ENST00000266087 and ENST00000382058), resulting from the usage of alternatively spliced 5′-exons, and encoding two FBXO7 protein isoforms of 522 and 443 amino acids (also referred to as isoform 1 and isoform 2), only differing at the N-terminus [10]. In the zebrafish genome, a single homologue to *hFBXO7*, here termed *zFbxo7*, is annotated (ENSDART00000082132). Its 1452-nucleotide transcript is predicted to encode a protein of 483 amino acids, which displays the same domain organization as the human longer FBXO7 isoform 1, and shows an overall 40% amino acid identity, rising to 65% identity and 78% similarity in the F-box domain (Figure 1A). The high level of sequence identity and similarity suggests functional conservation between zebrafish and human Fbxo7 proteins.

To confirm our *in silico* analyses, we amplified and sequenced the *zFbxo7* cDNA from the tupfel long fin (TL) zebrafish. This revealed that the *zFbxo7* transcript is the product of 10 exons, as also annotated in Ensembl. Compared with the sequence annotated in Ensembl (ENSDART00000082132) we only detected one polymorphic variant, a heterozygous insertion of two CAG triplets at position +448 from the A of the ATG start codon, leading to in-frame incorporation of two additional residues in a glutamine stretch.

### zFbxo7 protein expression throughout embryonic development and in adult tissues

The pattern of zFbxo7 protein expression was studied by Western blot (WB), using a mouse polyclonal antibody raised against full-length human FBXO7, which was previously validated by us for the human FBXO7 proteins in both WB and immunohistochemistry [10]. A single band corresponding to the predicted size of the zebrafish zFbxo7 protein was detectable at 12 hours post fertilization (hpf), which gradually increased in abundance during pharyngula, hatching stages, reaching a peak at the larvae stage (72 hpf) (Figure 1B). In 8-month-old adult wild type (WT) zebrafish, the zFbxo7 protein was abundantly expressed in the brain and liver, and hardly detected in the heart and kidney (Figure 1C). We further characterized the expression of the zFbxo7 protein in the brain by immunohistochemistry using the same antibody. The zFbxo7 immunoreactivity was ubiquitously present, more prominent in neurons of the olfactory bulb and diencephalon, intermediate in cerebellum and medulla oblongata, and weaker in the optic tectum and habenula (Figure 1D). The zFbxo7 immunoreactivity was prominent in the neuronal nuclei, but also present in the cytoplasm.

### Knock down of zFbxo7 results in developmental defects

**Figure 2:**
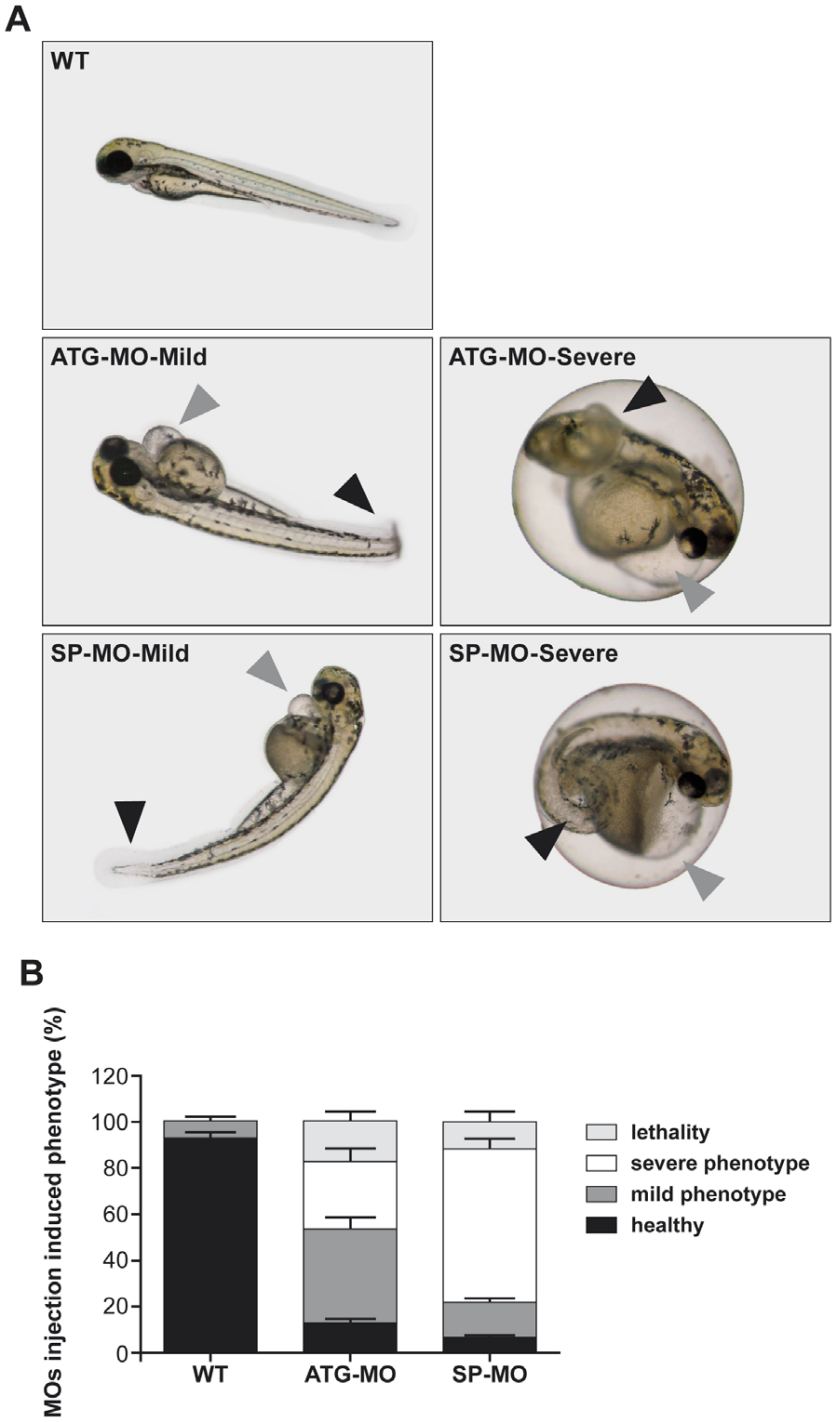
*zFbxo7* knock down results in developmental defects. (A) Representative images of zebrafish wild type and morphants. Injection of ATG-MO or SP-MO induced a range of phenotypes, which were grouped in mild and severe, including curly tails (black arrowheads), heart edema and heart malformations (grey arrowheads). (B) Percentages of healthy phenotype, mild phenotype abnormalities, severe phenotype abnormalities and lethality among uninjected control (WT) and MOs-injected morphants. *zFbxo7* knock down results in decreased zFbxo7 protein expression

Two non-overlapping *zFbxo7* MOs were designed, one targeting the ATG translation initiation site (ATG-start-codon-targeting MO, ATG-MO) and the other targeting the exon2/intron2 splice site (splice-site-targeting MO, SP-MO) of *zFbxo7*, respectively. The MOs were injected into the embryo yolk at one-cell or two-cell stage. No gross morphological abnormalities were observed at 24 hpf and 48 hpf in the MOs injected embryos, compared to non-injected ones (data not shown). A range of morphological phenotypes was observed at 72 hpf, including curly tails, heart edema, and heart malformations (Figure 2A). These phenotypes were similar in the morphants treated with ATG-MO and in those treated with SP-MO. The morphants were then divided in two groups, according to the severity of their phenotypes. Lethality was also quantified. The percentages of mild and severe phenotypes and of lethality associated with the injection of the ATG-MO and the SP-MO (N = 300 morphants for each of the two MO), are shown in the Figure 2B. Injection of the ATG-MO resulted in 17% lethality, 42% mild phenotype (ie. characterized by heart edema and slightly curly tail, ATG-MO-Mild), and 29% severe phenotype (ie. severe heart deformation and severe curly tail, ATG-MO-Severe). The morphants injected with SP-MO showed 12% lethality, 15% mild phenotype (SP-MO-Mild) and 67% severe phenotype (SP-MO-Severe).

**Figure 3:**
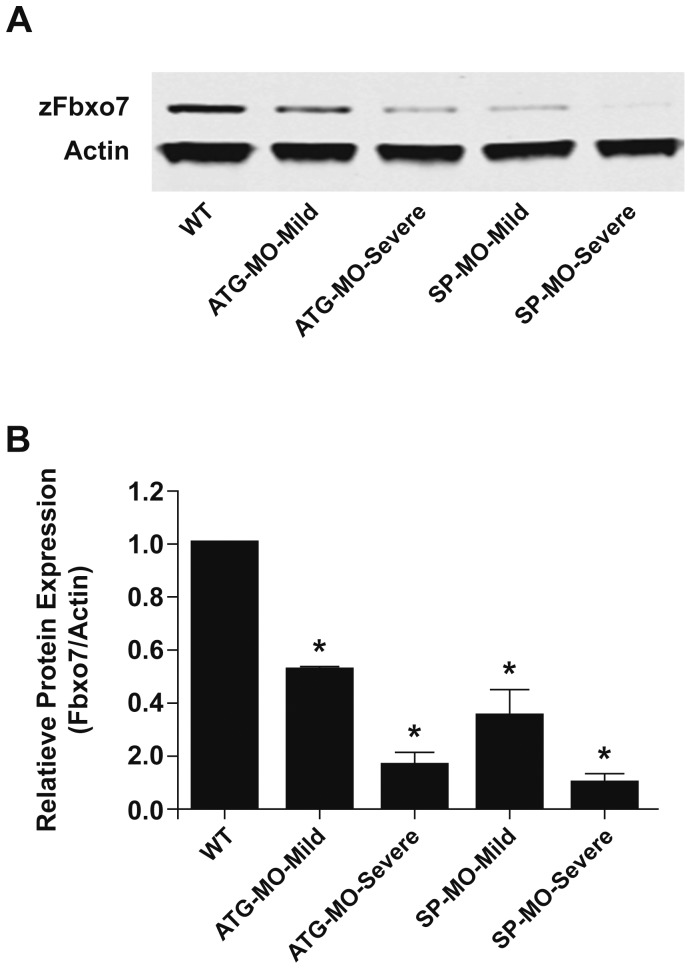
*zFbxo7* knock down results in decreased zFbxo7 protein expression. (A) Western blot of the zFbxo7 protein at 72 hpf in uninjected control (WT) and MOs-injected morphants which showed mild or severe phenotype abnormalities. (B) Quantification of the zFbxo7 protein levels is shown in panel A (Odyssey software). Data were collected from three independent experiments, *P*<0.01.

The efficiency of knock-down was monitored by measuring the zFbxo7 protein levels using western blot. Markedly and significantly decreased expression levels were observed after the injection of either MOs (Figure 3). Of note, the morphants displaying severe phenotypes showed more severe zFbxo7 protein depletion (to ∼16% of normal protein levels in the ATG-MO morphants, and to ∼10% of normal levels in SP-MO morphants), compared with those displaying mild phenotypes (∼53% of normal protein levels in the ATG-MO morphants, and ∼35% of normal levels in SP-MO morphants) (Figure 3B). This correlation between severity of protein depletion and severity of phenotype was present for both *zFbxo7*-targeting MO strategies. The lowest levels of zFbxo7 protein expression were seen in SP-MO-Severe morphants (Figure 3).

### *zFbxo7* knock down leads to abnormal patterning and dopaminergic neuronal cell loss

**Figure 4:**
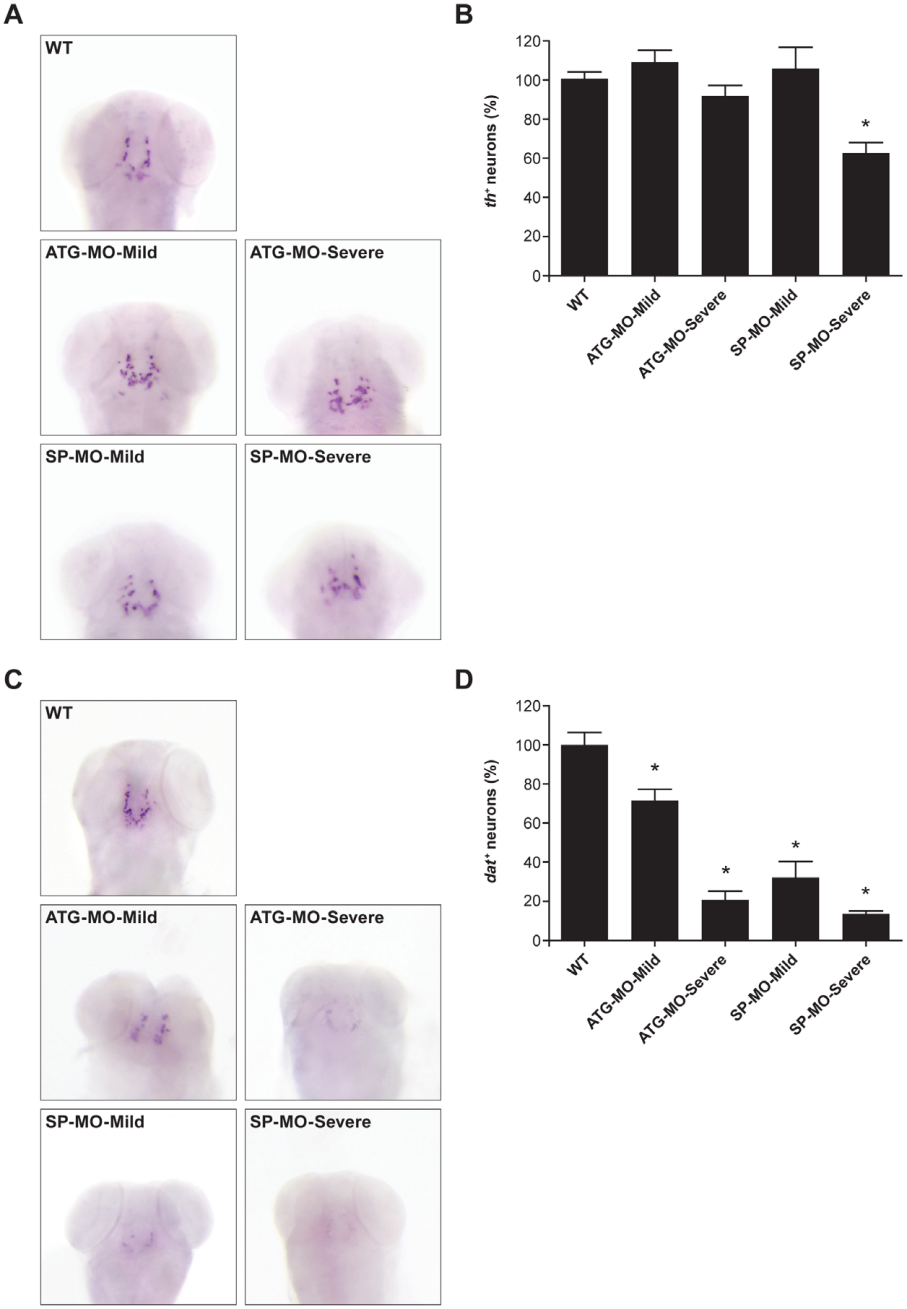
*zFbxo7* knock down results in dopaminergic neuronal cell loss. The brain catecholaminergic neurons were visualized by whole-mount *in situ* hybridization using antisense RNA probes specific for *tyrosine hydroxylase* (*th*, panel A) or *dopamine transporter* (*dat*, panel C). Number of neurons were counted manually and normalized to the counts in wild type zebrafish (panels B and D). * *P*<0.01

To investigate the effect of the zFbxo7 knock down on the development of the brain dopaminergic neurons, we studied the expression of the *tyrosine hydroxylase* (*th*), and the *dopamine transporter* (*slc6a3*, *dat*) mRNA, using whole mount *in situ* hybridization (WISH). Eighty morphants were analyzed in each experiment. In the zFbxo7 morphants, the patterning of the *th^+^*/*dat^+^* diencephalic dopaminergic neurons was disturbed (neurons were organized in more compact groups) compared to wild type animals (Figure 4A and 4C). Furthermore, a significant reduction (40%) in the number of *th^+^* neurons was seen, but only in the SP-MO-severe morphants compared with WT zebrafish (Figure 4A and 4B). On the contrary, the number of *dat^+^* neurons was significantly reduced in both the ATG-MO and SP-MO morphants, and more dramatically in the morphants with more severe phenotypes (ATG-MO-Severe and SP-MO-Severe) where the *dat^+^* neurons were hardly detectable (Figure 4C and 4D).

### *zFbxo7* knock down results in locomotor defects, which are improved by apomorphine

**Figure 5:**
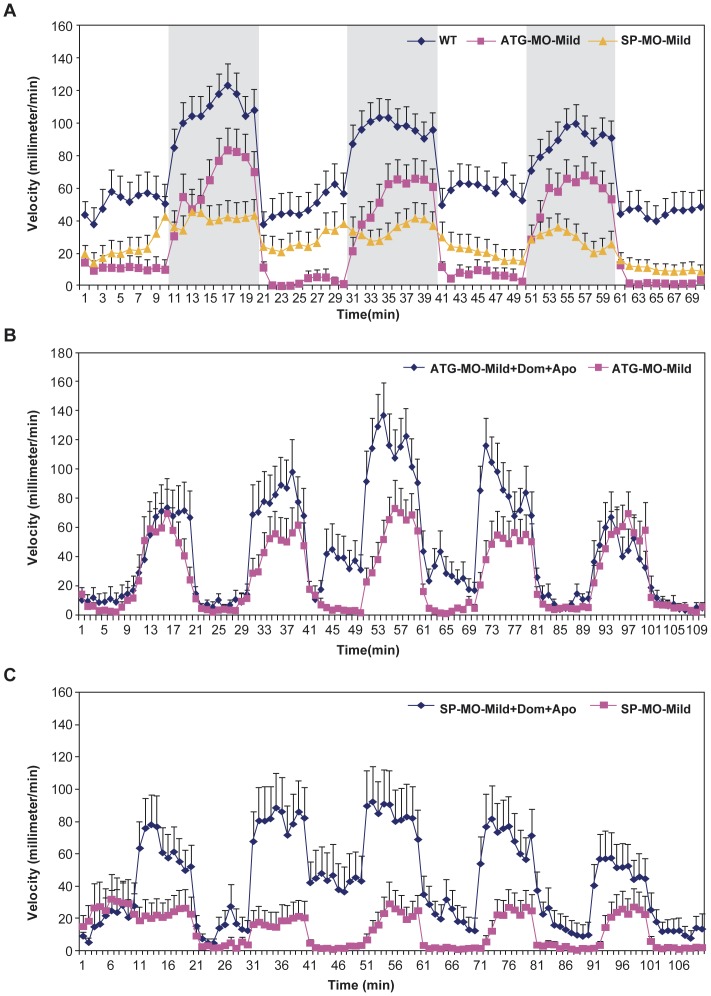
*zFbxo7* knock down results in locomotor defects, which are improved by apomorphine. The movements of WT zebrafish, ATG-MO-Mild and SP-MO-Mild morphants were recorded during three cycles of 10-minutes light/10-minutes darkness (periods of darkness are shown in grey). Compared with WT, morphants showed significantly decreased velocity in both light and dark phases (*P*<0.01, panel A), which were significantly improved by treatment with apomorphine (*P*<0.01 in the dark phase, panels B and C). Dom: domperidone. Apo: apomorphine.

ATG-MO-Severe and SP-MO-Severe morphants showed hardly any motor activities. Further locomotor analyses were therefore focused on the ATG-MO-Mild and SP-MO-Mild morphants. In order to assess the locomotor behavior, the swimming velocity was automatically measured in wild type zebrafish and morphants at 96 hpf, during cycles of light/darkness. Both the ATG-MO-Mild and SP-MO-Mild morphants displayed significantly decreased swimming velocity, compared to wild type zebrafish (Figure 5A).

In order to assess whether these locomotor defects are dependent by the dopamine deficiency in the brain (and not by general developmental delay), we studied the effects of apomorphine, a potent, direct dopamine agonist, also used in the treatment of PD patients. In order to prevent unwanted effects of apomorphine on the peripheral (extra-cerebral) dopamine receptors, we first exposed the animals to domperidone, a dopamine-receptor peripheral antagonist that does not cross the blood-brain barrier. Domperidone is also widely used in humans to prevent the peripheral side effects of apomorphine and other dopamine agonists (vomiting, hypotension).

We first show that no locomotor effects are detectable after placing either wild type zebrafish or morphants in water containing domperidone alone, at a concentration of up to 3 µM (Figure S2B and S2C). We then exposed wild type and morphants to water containing 3 µM domperidone and 3 µM apomorphine. While no effects were detectable in the wild type animals (Figure S2A), the swimming defects in the morphants were markedly and significantly improved, their performances reaching levels similar to those of the wild type animals (Figure 5B and 5C).

### Off-target effects due to MO-induced p53 activation are not detected

**Figure 6:**
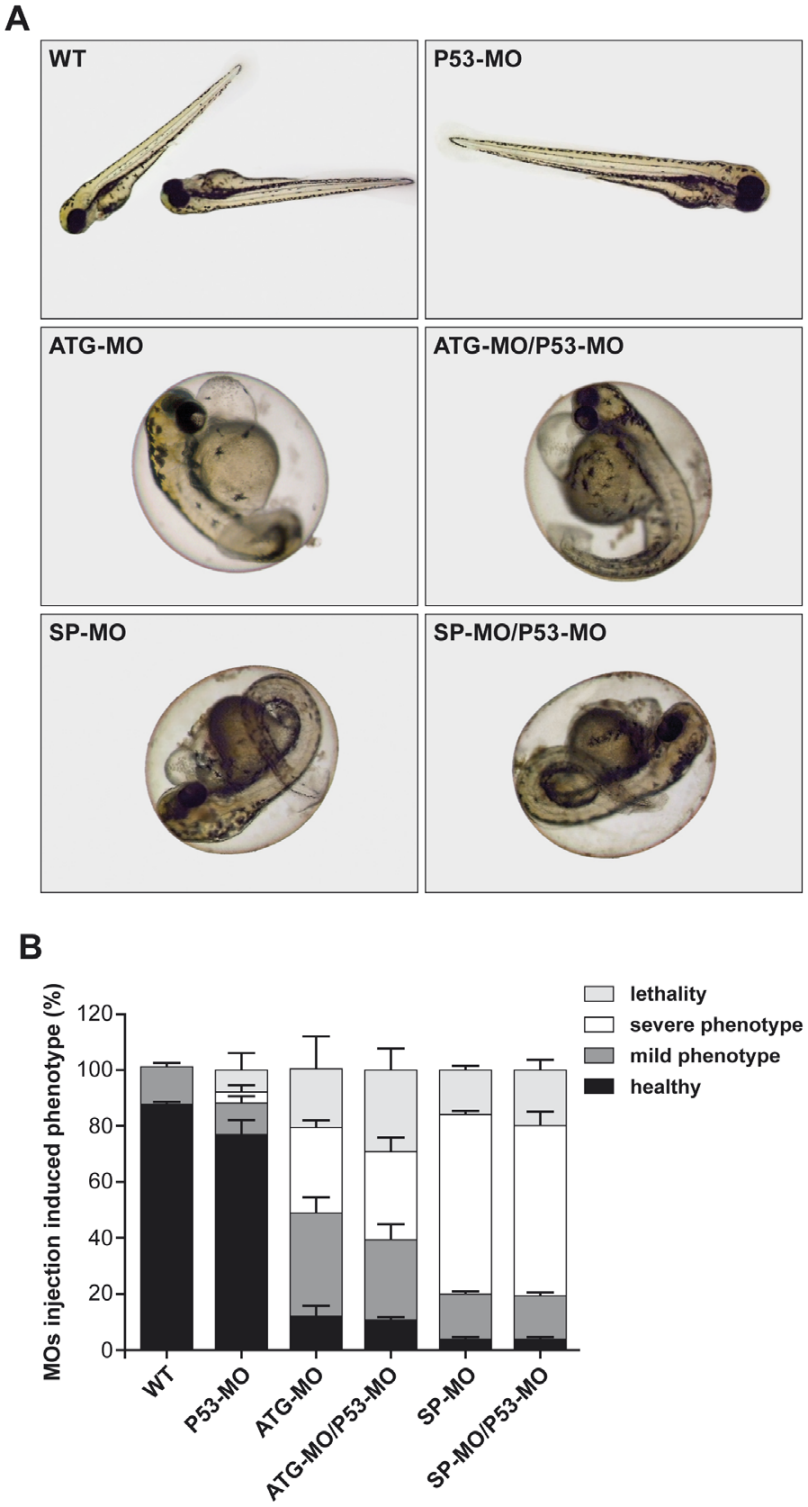
Off-target effects due to MO-induced p53 activation are not detected. (A) Representative images of zebrafish embryos treated with single MO injection (ATG-MO, SP-MO or P53-MO) or co-injection (ATG-MO/P53-MO or SP-MO/P53-MO). (B) Percentage of healthy phenotype, mild phenotype abnormalities, severe phenotype abnormalities and lethality among uninjected control (WT), single injected morphants and co-injected morphants.

To prevent off-target MOs effects due to activation of p53 expression, a specific *p53*-targeting MO was co-injected with *zFbxo7*-specific MOs (Figure 6A). The frequency of healthy phenotype, mild phenotype abnormalities, severe phenotype abnormalities, and lethality, among uninjected controls, single-injected morphants, and morphants co-injected with *p53*-targeting MO, was unchanged (Figure 6B). These results indicate that the observed phenotypes are not due to off-target effects mediated by the induction of p53 expression.

### Rescue of the phenotypes associated with *zFbxo7* MOs injection

In order to rescue the phenotypes associated to *zFbxo7* MOs injection, human *FBXO7* (*hFBXO7*) mRNA was prepared, and the quality confirmed by *in vitro* translation (Figure S1A). The hFBXO7 protein is ∼5 kDa larger than the zFbxo7 protein, and therefore, these two proteins can be easily distinguished by Western blot. However, when co-injected together with *zFbxo7* MOs, the *hFBXO7* mRNA failed to rescue the morphological phenotypes induced by *zFbxo7* MOs (data not shown). The analysis of the temporal pattern of expression showed strong levels of the exogenous hFBXO7 protein at 8 hpf, which quickly wore off and disappeared at 48 hpf (Figure S1B). On the contrary, the levels of the endogenous zFbxo7 protein were almost undetectable before 12 hpf, and increased gradually until 72 hpf (Figure 1B). The discrepancy between the time course of expression of the exogenous hFBXO7 and the endogenous zFbxo7 protein (which could be due to the usage of different promoters) is likely the explanation of the lack of rescuing effects.

## Discussion

PARK15 is an autosomal recessive disease, caused by the loss of function of the proteins encoded by the *FBXO7* gene [7], and in particular, by the loss of the function of the longer FBXO7 isoform (isoform 1), which localizes in the cell nucleus [10]. However, how the loss of this function leads to neurodegeneration with massive, early death of the nigrostriatal dopaminergic neurons remains unknown. Here, we establish the first zebrafish model of PARK15, by transient knock down of the *zFbxo7* expression using MOs. We show that two different, non-overlapping *zFbxo7* MOs are able to efficiently knock down the zFbxo7 protein expression, resulting in developmental defects, abnormalities at the level of the patterning and number of the brain dopamine neurons, and locomotor defects. These last defects are dramatically improved by the dopamine agonist apomorphine.

Tyrosine hydroxylase (Th), the rate-limiting enzyme in the synthesis of dopamine and other catecholamines, is expressed by all the catecholaminergic neurons (dopaminergic, noradrenergic and adrenergic) [13], [14]. On the contrary, the dopamine transporter (Dat) is a specific dopaminergic neuronal marker [15], [16]. In wild type zebrafish, diencephalic clusters of *th^+^*/*dat^+^* dopaminergic neurons are present, which project to the ventral telencephalon, and might be functionally equivalent to human nigrostriatal or ventral tegmental dopaminergic neurons (Figure 4A and 4C) [17], [18].

Th or Dat staining at the level of mRNA (WISH) or protein (immunohistochemistry) are widely used to assess the number of dopaminergic neurons in animal models of PD, including the zebrafish (reviewed in [11], [12]). Zebrafish knock down models have been previously generated targeting the homologues of other PD-causing genes, including *PARKIN* [19], [20], *PINK1* [21], [22], [23], *DJ-1* [24], [25], and *LRRK2* [26], [27], while the *alpha-synuclein gene* has no homologue in zebrafish [28]. Overall, the results of these studies have not been very consistent, depending in part from the different knock out strategies and efficiencies. Also, off-target effects were not entirely excluded in some studies reporting the most severe phenotypes [21]. Of note, none of the previous models has shown robust evidence of dopaminergic neuronal loss, together with, dopamine-dependent behavioral abnormalities. Instead, the zFbxo7 knock down model described here yields abnormalities at the level of both patterning and number of the brain dopamine neurons, as well as dopamine-dependent locomotor defects.

We show that the depletion of zFbxo7 alters the patterning of the *th^+^*/*dat^+^* diencephalic dopaminergic neurons (Figure 4A and 4C), suggesting an important role for the zFbxo7 protein in the development of the brain dopaminergic systems. A similar disturbance of dopaminergic neuronal patterning, without neuronal loss, has been reported after the knock down of the zebrafish homologue of human *PINK1* [23].

We also show that the number of *th* ^+^ neurons is significantly reduced (by 40%), in the morphants with most severe phenotypes, and using the SP-MO morpholinos (Figure 4A and 4B). Similar loss of *th* ^+^ neurons (∼40%) was previously reported in one knock down model of the zebrafish *PINK1* homologue [21], though off-target effects were not excluded, and subsequent studies did not report that phenotype [22], [23], [29]. A milder (∼20%) loss of *th* ^+^ neurons was reported in one knock down model of the zebrafish *PARKIN* homologue, in absence of behavioral abnormalities [19], while neither loss of *th* ^+^ neurons nor behavioral phenotypes were reported in a second study using a different knock down strategy for the *PARKIN* zebrafish homologue [20]. Loss of *th* ^+^ neurons was not reported in two knock down studies of the zebrafish *DJ-1* homologue [24], [25], while they were detected in only one of two knock down studies of the zebrafish *LRRK2* homologue [26], [27].

Of note, the *dat* expression was not investigated in most of these previous studies, which only used *th* expression to assess the integrity of the dopaminergic neurons. Intriguingly, we found that the number of *dat^+^* neurons was reduced much more than the number of the *th^+^* neurons, in both the ATG- and SP-MOs morphants, and even more dramatically in the morphants with more severe phenotypes (Figure 4C and 4D). This discrepancy between the reduction in the *th^+^* and *dat^+^* neurons might indicate, in part, a selective loss of dopaminergic neurons, within the larger compartment of the catecholaminergic neurons. However, this pattern more likely indicates the presence of surviving *th^+^* dopaminergic neurons with down-regulated *dat* expression. The same pattern (reduced DAT in preserved DA neurons) is seen *in vivo* in humans with genetic forms of PD by PET imaging in the earlier stages of dopaminergic neuronal degeneration. This might represent a compensatory neuronal reaction that maintains sufficient synaptic dopamine levels by down-regulating the DAT-mediated presynaptic reuptake of the neurotransmitter [30]. Besides the dopaminergic neurons, we cannot exclude that other neuronal populations were affected in the brain or the spinal cord of these morphants, as this was not specifically addressed here.

As a behavioral correlate, there are marked locomotor defects in the morphants injected with both *zFbxo7* MOs, and more importantly, these defects are dramatically improved by a direct, centrally-acting dopamine agonist, apomorphine. Of note, we prevented the effects of apomorphine on the peripheral dopamine receptors by co-administering domperidone, a dopamine-receptor antagonist that does not cross the blood-brain barrier. In these conditions, the observed effects are due to the action of apomorphine on dopamine receptors within the brain. The dopamine-dependence of the locomotor defects (bradykinesia) is a hallmark of PARK15 and PD in general, and it indicates the presence of presynaptic lesions at the level of the nigrostriatal dopaminergic neurons, in the context of preserved post-synaptic dopamine receptor and downstream brain circuitry. This hallmark feature is thus reproduced in the zebrafish *fbxo7* model.

The specificity of MO-mediated gene knock down is an important issue in zebrafish models. In this study, we used two non-overlapping MOs, targeting the ATG start codon and the intron2/exon2 splice site of *zFbxo7*, respectively. Both of them resulted in efficient zFbxo7 depletion (measured by western blot), and qualitatively similar neuronal phenotypes. This is the first evidence that the effects are due to the specific knock down of the zFbxo7 protein. Furthermore, the degree of zFbxo7 protein depletion correlated with the severity of embryonic development defects, of neuronal abnormalities and of the locomotor phenotypes. This is a second argument in support that the observed phenotypes are specifically due to the depletion of the zFbxo7 protein. It is well-known that the injection of MO might induce apoptosis by activating the expression of the *p53* transcription (general MO toxicity) [31], [32]. Therefore, we also excluded that the observed phenotypes were caused by the activation of p53, by co-injecting *p53*-targeting MOs.

Another way to support specificity of effects would be rescuing these phenotypes by using the mRNA of the specific gene of interest. Unfortunately, here *hFBXO7* failed to rescue the morphological phenotypes induced by *zFbxo7* MOs. However, a detailed analysis of the temporal pattern of expression disclosed a clear discrepancy between the time course of expression of the exogenous hFBXO7 and the endogenous zFbxo7 protein. Moreover, it is very difficult to reproduce the cell-specific expression pattern of endogenous proteins by overexpressing exogenous proteins. The timing and localization of Fbxo7 expression might therefore be critical to its function, and the early and short-lasting expression of the *hFBXO7* mRNA is likely the explanation of the lack of rescuing effects. Lack of rescue of truly-specific effects is a well-known phenomenon in zebrafish MO-mediated modeling [11], [12].

We acknowledge a more general caveat in modeling late-onset human neurodegenerative diseases, such as PD, by transient gene knock down during the embryonic development of a model organism. Indeed, besides a clear PD-related phenotype, our zFbxo7 morphants also displayed overt developmental abnormalities outside the brain, including heart malformations and curly tails that are not features of the human pathology. PD manifests as a post-developmental phenotype, even in the early-onset cases like those related to FBXO7 deficiency.

In conclusion, this novel vertebrate model reproduces pathologic and behavioral hallmarks of human parkinsonism (dopaminergic neuronal loss and dopamine-dependent bradykinesia), representing therefore a valid tool for investigating the mechanisms leading to selective dopaminergic neuronal death, screening for modifier genes or libraries of potential therapeutic compounds.

## Materials and Methods

### Zebrafish maintenance

The use of zebrafish embryos for this study was approved by the Institutional Review Board for experimental animals of the Erasmus MC, Rotterdam. All procedures and conditions were in accordance with Dutch animal welfare legislation. Wild type tupfel long fin zebrafish were used for all experiments. Embryos were collected after natural spawning and raised in embryo medium containing methylene blue at 28°C under standard conditions [33].

### Genetic analysis of the zebrafish *FBXO7* orthologue

The sequences of the *zFbxo7* transcript and protein (ENSDART00000082132, ENSDARP00000076569) were retrieved from Ensembl, and the zFbxo7 protein was blasted to the human FBXO7 proteins, isoform 1 (ENSP00000266087) and isoform 2 (ENSP00000371490). Total RNA was isolated from 72 hpf tupfel long fin zebrafish as described before [10], and complementary DNA was synthesized using the iScript™ cDNA Synthesis Kit (Bio-Rad) according to the manufacturer's instructions. The coding region of *zFbxo7* was amplified and sequenced (PCR primers are shown in the Supplementary Material, Table S1), and aligned to the sequence deposited in Ensembl (ENSDART00000082132).

### Western blot

Zebrafish embryos at different developmental stages, as well as different organs of eight-month-old adult zebrafish were collected, and the proteins extracted by homogenization with buffer containing 10 mM HEPES, 300 mM KCl, 3 mM MgCl_2_·6H_2_O, 100 µM CaCl_2_·2H_2_O, 0.45% Triton X-100 and 0.05% Tween-20, pH 7.6. Thirty µg of total protein were separated in 6%–12% Criterion™ XT 4–12% Bis-Tris Gel (Bio-Rad), and blotted with nitrocellulose membrane as previously described [10]. The primary antibodies used were: mouse polyclonal antibody raised against full-length human FBXO7 (Abnova, 1/3000), and mouse monoclonal anti-β-Actin (Sigma, 1/10000). After incubation with secondary antibody, the membrane was scanned with the Odyssey TM Infrared Imager (Li-COR Biosciences). The integrated intensities of the zFbxo7 protein bands were quantified by the Odyssey software, using Actin as loading control.

### Immunohistochemistry

The brain of eight-month-old zebrafish was dissected and fixed in 4% phosphate-buffered paraformaldehyde (PFA) overnight. Paraffin embedded sections (6 µm) were prepared for immunostaining. Briefly, dewaxed sections were pretreated for antigen retrieval by microwave heating in 0.1 M sodium citrate buffer (pH 6). Immunostaining was performed with mouse polyclonal antibody raised against full-length human FBXO7 (Abnova, 1/40) followed by indirect immunoperoxidase labeling and hematoxylin counterstain.

### Morpholino and mRNA microinjections

Anti-sense morpholinos (MOs) were purchased from Gene Tools LLC (Philomath OR). Two MOs were designed to target *zFbxo7*: one was targeting the *zFbxo7* translation initiation site (**ATG-MO**, 5′-GAG CTT CAT TCT GTG CTT CCA GAA A-3′), and another for the *zFbxo7* exon2/intron2 splice site (**SP-MO**, 5′-GAT GAA GGT GCT CAG ACT GAC CGG A-3′). A previously described MO targeting the translation initiation site of *p53* (**P53-MO**, 5′-GCG CCA TTG CTT TGC AAG AAT TG-3′) was also used [32].

All MOs were dissolved in double distilled H_2_O and diluted with Danieau solution (58 mM NaCl, 0.7 mM KCl, 0.4 mM MgSO_4_, 0.6 mM Ca(NO_3_)_2_, 5.0 mM HEPES pH 7.6), containing 1% phenol red as indicator. The amounts of ATG-MO and SP-MO were optimized for maximal knock-down efficiency and minimal toxicity (data not shown), and 4 ng of ATG-MO and 8 ng of SP-MO were selected for the following experiments. These MO were injected into the embryos yolk at one-cell or two-cell stage to knock down the expression of *zFbxo7*. In separate experiments, the P53-MO was co-injected with *zFbxo7*-specific MO (6 ng with ATG-MO or 8 ng with SP-MO), to prevent off-target effects due to activation of p53 expression.

Full length human *FBXO7* cDNA (*hFBXO7*) amplified by RT-PCR from peripheral mononuclear blood cells [10] was ligated into the pCR2.1-TOPO vector (Invitrogen), and subcloned at the site of *EcoR*I in pCS2+ vector [34]. The fidelity of *hFBXO7*-pCS2 was verified by direct sequencing. Using *Not*I-linearized h*FBXO7*-pCS2 as template, *hFBXO7* mRNA was generated with the mMessage mMachine SP6 kit (Ambion). To test the quality of h*FBXO7* mRNA, the *in vitro* translation was performed using Rabbit Reticulocyte Lysate System (Promega). For rescue experiments, *hFBXO7* mRNA was co-injected with *zFbxo7* MOs at one cell stage. In all the experiments, the morphology of morphants was observed at 24, 48, and 72 hpf by two investigators in blind conditions.

### Whole mount *in situ* hybridization

Briefly, a digoxigenin-labelled antisense RNA probe specific for the *tyrosine hydroxylase* (*th*) transcript was synthesized from linearized pCRII-TOPO-*th* plasmid and transcribed by T7 RNA polymerase (Roche). The plasmid containing the *dopamine transporter* (*dat*, *slc6a3*) transcript was a kind gift from Dr. Edward A. Burton, Department of Neurology, University of Pittsburgh School of Medicine, Pittsburgh, PA, (USA) [35], and the corresponding RNA probe was generated by T3 RNA polymerase (Roche). Embryos were fixed overnight at 72 hpf in 4% PFA, and bleached with 10% H_2_O_2_ to remove pigmentation. Embryos were then transferred to 100% methanol for dehydration at −20°C for at least 24 h and then the hybridization procedure was followed as previous described [36]. After staining with NBT/BCIP solution (Roche), labeled embryos were washed with PBST (0.1% Tween 20 in PBS) in dark and mounted with 80% glycerol. The images of *th* ^+^ and *dat* ^+^ neurons were acquired and quantified by two investigators in blind fashion, under an Olympus microscope. The results are shown as percentages of the labeled neurons present in uninjected wild type embryos, staged and treated in parallel with the *zFbxo7* knock-down morphants.

### Locomotor activity studies

For behavioral studies, wild type and morphant larvae were harvested at 96 hpf, and placed in 96-well plates (one larva per well) containing 150 µl of embryo medium at 28°C. The larvae were allowed to acclimatize for 15 min before starting the behavioral monitoring. DanioVision (Noldus) was used for tracking movement during three cycles of 10-min white light-on (light) and 10-min light-off (darkness). All digital tracks were analyzed by Ethovision XT software (Noldus) for velocity, and a minimum movement distance of 0.2-mm was used to filter out system noise.

For the assessment of the dopamine-dependence of the locomotor defects, morphants were first kept for one hour in water containing 3 µM domperidone, an orally active compound that blocks the peripheral dopamine receptors, but does not cross the blood-brain barrier. At this concentration, domperidone induced no behavioral effects in wild type or in morphants. After the pre-treatment with domperidone, the larvae were placed in water containing 3 µM apomorphine (a potent dopamine receptor full agonist), and the swimming activity was tracked during five cycles of light-on/light-off.

### Data analysis

Quantitative data are expressed as means ± SEM, and each treatment group was normalized to the wild type control group. All experiments were done in triplicates, and the statistical analyses were performed using one-way ANOVA or T-test, as appropriate, with the SPSS package. The data were considered statistically significant at *P*<0.01.

## Supporting Information

Figure S1 **Expression of endogenous zFbxo7 and exogenous hFBXO7 occurs in different time points during the zebrafish development.**(A) Western blot analysis after *in vitro* protein translation of the *hFBXO7* mRNA. A band of the expected size of the hFbxo7 protein was detected, validating the *hFBXO7* mRNA as a rescuing template mRNA. An empty lane (Control) shows the reaction product after omitting the *hFBXO7* mRNA template. (B) Time course of the expression of exogenous hFBXO7 *in vivo* in wild type embryos.The *hFBXO7* mRNA was injected into one-cell stage embryos, and the expression of hFBXO7 was probed at 8, 24 and 48 hpf by Western blot. The expression of hFBXO7 was already markedly lower at 24 hpf, and was undetectable at 48 hpf.(C) The expression of exogenous hFBXO7 and endogenous zFbxo7 *in vivo* in zebrafish embryos with or without co-injection of SP-MO. The *hFBXO7* mRNA and/or SP-MO were injected into one-cell stage embryos, and the expression of proteins was probed at 8 and 72 hpf by Western blot. The expression of the endogenous zFbxo7 was maximal at 72 hpf, when the exogenous hFBXO7 was undetectable.
(TIF)

Figure S2 **Locomotor behavior is not affected by domperidone.**The automated analysis of locomotion shows that the treatment with domperidone together with apomorphine (A) or domperidone alone (B) induced no detectable effects on the wild type zebrafish. Furthermore, domperidone alone induced no detectable locomotor effects in the ATG-MO-injected morphants (C). Dom: domperidone. Apo: apomorphine.
(TIF)

Table S1 PCR primers used for the amplification of the *zFbxo7* cDNA.
(PDF)
