# Supplementary figures and images for: Dopaminergic Neuronal Loss and Dopamine-Dependent Locomotor Defects in Fbxo7-Deficient Zebrafish
Source: PLoS One. 2012 Nov 2;7(11):e48911. doi: 10.1371/journal.pone.0048911 (PMC3487786; doi:10.1371/journal.pone.0048911)

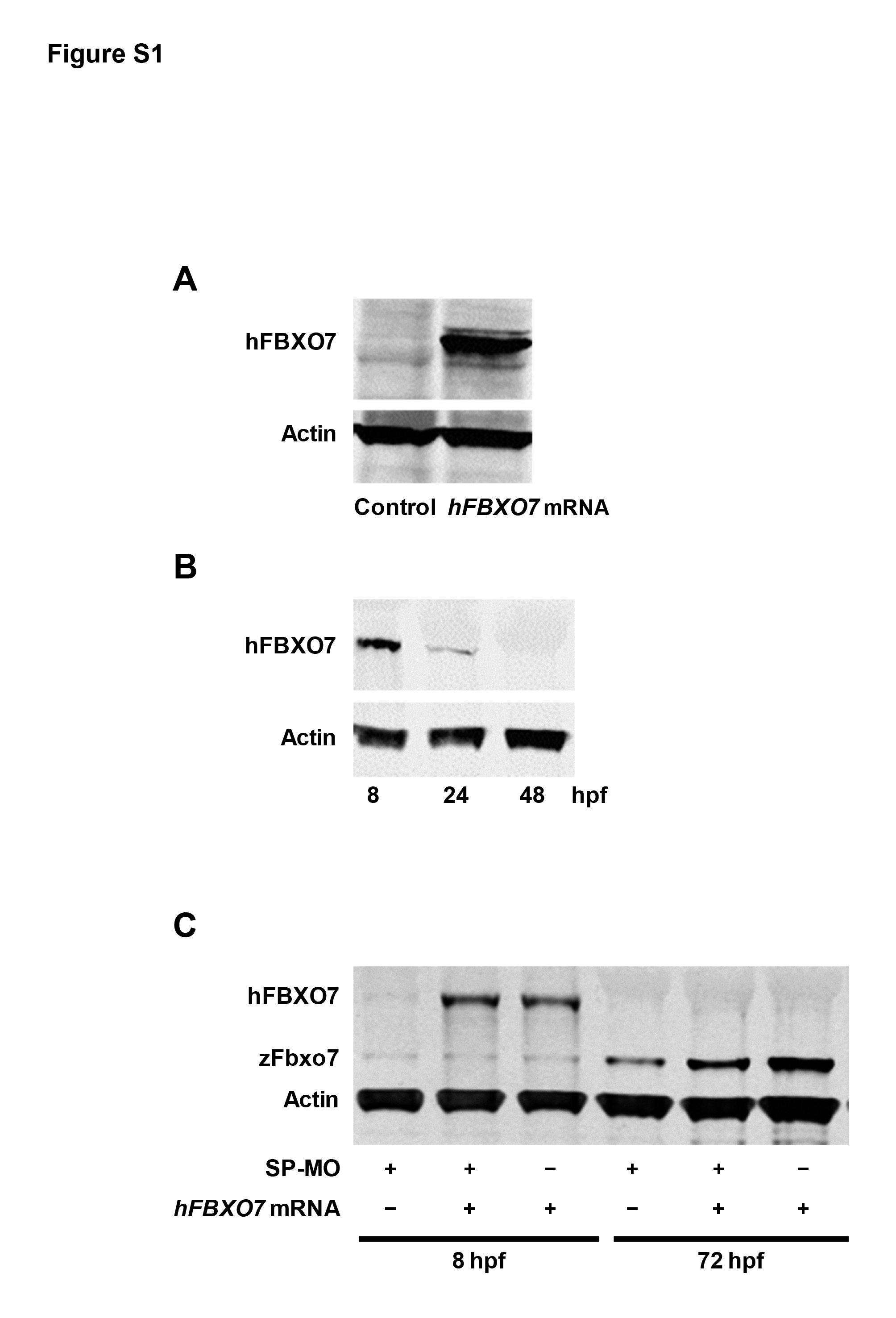

Supplement: Figure S1 — Expression of endogenous zFbxo7 and exogenous hFBXO7 occurs in different time points during the zebrafish development.(A) Western blot analysis after in vitro protein translation of the hFBXO7 mRNA. A band of the expected size of the hFbxo7 protein was detected, validating the hFBXO7 mRNA as a rescuing template mRNA. An empty lane (Control) shows the reaction product after omitting the hFBXO7 mRNA template. (B) Time course of the expression of exogenous hFBXO7 in vivo in wild type embryos.The hFBXO7 mRNA was injected into one-cell stage embryos, and the expression of hFBXO7 was probed at 8, 24 and 48 hpf by Western blot. The expression of hFBXO7 was already markedly lower at 24 hpf, and was undetectable at 48 hpf.(C) The expression of exogenous hFBXO7 and endogenous zFbxo7 in vivo in zebrafish embryos with or without co-injection of SP-MO. The hFBXO7 mRNA and/or SP-MO were injected into one-cell stage embryos, and the expression of proteins was probed at 8 and 72 hpf by Western blot. The expression of the endogenous zFbxo7 was maximal at 72 hpf, when the exogenous hFBXO7 was undetectable. (TIF) [file pone.0048911.s001.tif]

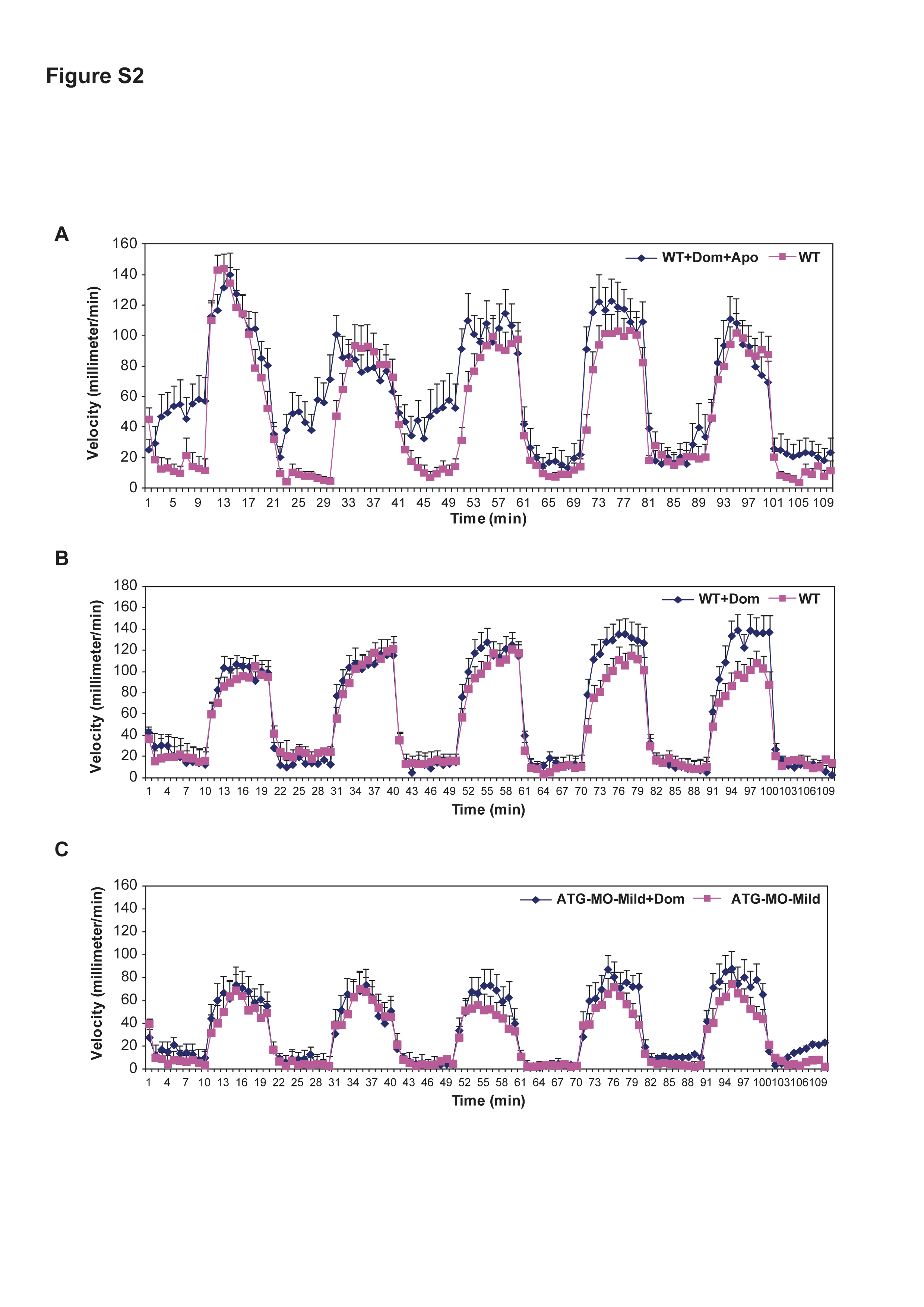

Supplement: Figure S2 — Locomotor behavior is not affected by domperidone.The automated analysis of locomotion shows that the treatment with domperidone together with apomorphine (A) or domperidone alone (B) induced no detectable effects on the wild type zebrafish. Furthermore, domperidone alone induced no detectable locomotor effects in the ATG-MO-injected morphants (C). Dom: domperidone. Apo: apomorphine. (TIF) [file pone.0048911.s002.tif]
