# Supplementary material for: Dopaminergic Neuronal Loss and Dopamine-Dependent Locomotor Defects in Fbxo7-Deficient Zebrafish
Source: PLoS One. 2012 Nov 2;7(11):e48911. doi: 10.1371/journal.pone.0048911 (PMC3487786; doi:10.1371/journal.pone.0048911)
Supplement: Table S1 — PCR primers used for the amplification of the zFbxo7 cDNA. (PDF) [file pone.0048911.s003.pdf]

Supplementary table S1

PCR primers used for the amplification of the *zFbxo7* cDNA

Exon	Forward primer (5’→3’)	Reverse primer (5’→3’)
1, 2, 3	ACTGCGTTACTTTGACGTTTCTG	TGCTGCTGCTGCTGATCC
4	CGTCGTCTCTGGTGATCTG	CTCCAGCAGAGGGTGGACA
5, 6	TGCTCTGCTGTGAGGCTG	CAAACGCAGCAGCAGCTCA
7, 8, 9	CCTGACGAGTATGTGACAGC	TGGCCGAGGAAGGATGAC
10	TCGATCTCTCGTCTCGCT	GAGGAGAAGCAGGCTTGAC
